# Case report: Ultrasound biomicroscopy as a guide for the selection of injection sites for dexamethasone intravitreal implant (Ozurdex) for peripheral granulomatous ocular toxocariasis in children

**DOI:** 10.3389/fmed.2023.1176585

**Published:** 2023-05-16

**Authors:** Xin Zhang, Xinzhu Hou, Yan Zhang, Jingjie Liu, Zhiyong Zhang

**Affiliations:** ^1^The Second School of Clinical Medicine of Zhejiang Chinese Medical University, Hangzhou, Zhejiang, China; ^2^Eye Center, Second Affiliated Hospital, School of Medicine, Zhejiang University, Hangzhou, Zhejiang, China

**Keywords:** ultrasound biomicroscopic, peripheral granulomatous ocular toxocariasis, pediatric uveitis, dexamethasone implant, Ozurdex, intravitreal injection, treatment

## Abstract

**Purpose:**

This article aims to report a case of successful treatment of peripheral granulomatous ocular toxocariasis (OT) in an 8-year-old patient using intravitreal injection of dexamethasone (DEX) implant (Ozurdex) under ultrasound biomicroscopy (UBM) guidance.

**Case presentation:**

A previously healthy 8-year-old boy with a history of long-term close contact with dogs complained of blurring of vision in the right eye for a year. Ophthalmic examination of his right eye showed chronic uveitis. Notably, UBM examination identified granulomas and peripheral vitreous strand in the ciliary body from 3 to 8 o'clock positions. Enzyme-linked immunosorbent assay (ELISA) results of the intraocular fluid (IF) and serum showed increased anti-Toxocara immunoglobulin G (IgG) levels, leading to a diagnosis of peripheral granulomatous OT in the right eye. Intraocular surgery was not indicated in this case. The treatment goal was to alleviate uveitis, improve visual acuity, and prevent complications. He was treated with an intravitreal injection of DEX implant, administered as a single dose every three months, total two doses, combined with albendazole, an oral anthelmintic. Under preoperative UBM guidance, two injections were performed at the 12 and 10 o'clock positions in the pars plana where there were no granulomas and peripheral vitreous strand, successfully preventing complications associated with intravitreal injection. After two injections, the patient's right eye vision improved significantly, with the best-corrected visual acuity (BCVA) increasing from 20/400 to 20/50. Vitreous opacity and retinal edema were reduced, preretinal proliferative membrane was stabilized, and no adverse events occurred.

**Conclusion:**

UBM can accurately determine the location and extent of peripheral granulomas in OT patients, facilitating the avoidance of granulomas during intravitreal injection and preventing complications associated with intravitreal injection. Under the close follow-up and strict adherence to indications, preoperative UBM-guided intravitreal injections of DEX implant treatment for pediatric peripheral granulomatous OT are safe and effective, providing a new therapeutic option for pediatric peripheral granulomatous OT.

## 1. Introduction

Ocular toxocariasis (OT) is a common yet often overlooked zoonotic parasitic infection of the eye, mainly manifesting as unilateral uveitis. The infection route is typically associated with contact with infected dogs or cats, or the consumption of food contaminated by their feces. Upon entering the small intestine, the eggs of *Toxocara canis* or *Toxocara cati* develop into second-stage larvae, which penetrate the intestinal wall and reach the uveal tract through blood circulation. Ocular lesions may result from direct injury by the larvae, indirect effects of larval toxic products, or host immune responses against the parasite (1, 2). OT most commonly occurs in children, particularly those from rural areas with poor sanitary conditions and frequent contact with dogs and cats (3–7). The three most common clinical manifestations of OT are peripheral granulomatous type, posterior pole granulomatous type, and vitreous opacities resembling uveitis (8). Complications such as cataracts, severe vitreous opacities, cystoid macular edema, tractional retinal detachment, and retinal damage caused by granuloma result in vision loss in patients with OT (9). Enzyme-linked immunosorbent assay (ELISA) can detect elevated levels of anti-Toxocara immunoglobulin G (IgG) in serum and intraocular fluid (IF) to confirm OT (10, 11). Empirical treatment of OT includes long-term oral steroid drugs combined with anthelmintic drugs, while pars plana vitrectomy (PPV) may be a more appropriate treatment for patients with macular epiretinal membrane, traction or rhegmatogenous retinal detachment, and severe or persistent vitreous opacity (1).

Peripheral granulomatous OT accounts for ~44 to 60% of all OT cases, with lesions ranging from the equator to the ora serrata. Patients' initial presenting visual acuity is often between 20/70 and 20/200. Lesion characteristics include dense granulomas or snowbank-like changes visible in the pars plana of the retina. Common clinical manifestations are posterior corneal deposits, iris adhesion, strabismus, moderate-to-severe vitreous inflammation, vitreous strands, tractional retinal detachment, and epiretinal membranes (12). Given the high prevalence of peripheral granulomatous OT and its impact on patients' visual acuity, we should pay more attention to this type of OT, strengthening diagnostic and therapeutic strategies to improve patients' visual acuity and quality of life.

The dexamethasone intravitreal implant (DEX implant, Ozurdex, Allergan Inc, Irvine, CA, USA) is a rod-shaped biodegradable implant that continuously releases dexamethasone (DEX) within the vitreous cavity (13). The US Food and Drug Administration (FDA) has approved DEX implant for the treatment of diabetic macular edema (DME), retinal vein occlusion (RVO), and non-infectious intermediate, posterior, and pan-uveitis (14, 15). The DEX implant possesses potent anti-inflammatory and immunosuppressive activities, effectively reducing inflammation and improving visual acuity (15, 16). It has been proven safe and effective in the treatment of pediatric non-infectious uveitis (17). Therefore, the DEX implant may serve as an alternative to systemic steroid drugs for the treatment of OT.

Ultrasound biomicroscopy (UBM) is a non-invasive ophthalmic diagnostic tool suitable for high-resolution imaging of anterior segment structures, such as the cornea, aqueous humor, iris, ciliary body, lens, and choroid (18–24). For pediatric patients who cannot provide detailed medical history or cooperate with examinations, UBM is the only option for achieving high-resolution, non-invasive imaging of the anterior segment (18, 25, 26). Given the capabilities of UBM, its use as an auxiliary examination tool can aid in the diagnosis and differential diagnosis of peripheral granulomatous OT. UBM has advantages over fundus photography, as it can detect hidden granulomas that are usually located within vitreous strands and on the ciliary body surface, particularly surrounding the ciliary crown (27). Through UBM, the location and extent of granulomas in the ciliary body, pars plana, and peripheral retina in OT patients can be determined (27). Furthermore, UBM helps in observing complications involving the peripheral retinochoroidal structures in OT patients, such as ciliary detachment (27, 28). Prior to surgery, UBM examination can assist surgeons in selecting appropriate PPV incision sites, avoiding contact with peripheral granulomas and preventing granuloma perforation-induced retinal break or retinal detachment (25). Therefore, UBM holds significant value in the diagnostic assessment and surgical guidance of peripheral granulomatous OT in children.

In this case report, we present the treatment process of an 8-year-old patient with peripheral granulomatous OT. Instead of conventional oral steroid drugs, the patient received an intravitreal injection of the DEX implant every 3 months (a total of two doses). Before treatment, we used UBM guidance to determine the location and extent of the granulomas to assist the surgeon in selecting the appropriate injection site. The purpose of this case report is to emphasize that by adopting a treatment plan of preoperative UBM-guided intravitreal DEX implant injections, we successfully controlled the uveitis in the OT patient, improved his visual acuity, and successfully avoided complications associated with intravitreal injection without any adverse reactions.

## 2. Case report

On July 9, 2021, an 8-year-old boy complaining of blurred vision in his right eye over the past year was admitted to the Second Affiliated Hospital of Medical College of Zhejiang University. The patient had no ocular redness, pain, or other discomforts. His parents reported that the patient had been in good health and delivered term without a history of oxygen supplementation. His family members had no symptoms of blurred vision.

**Figure 1:**
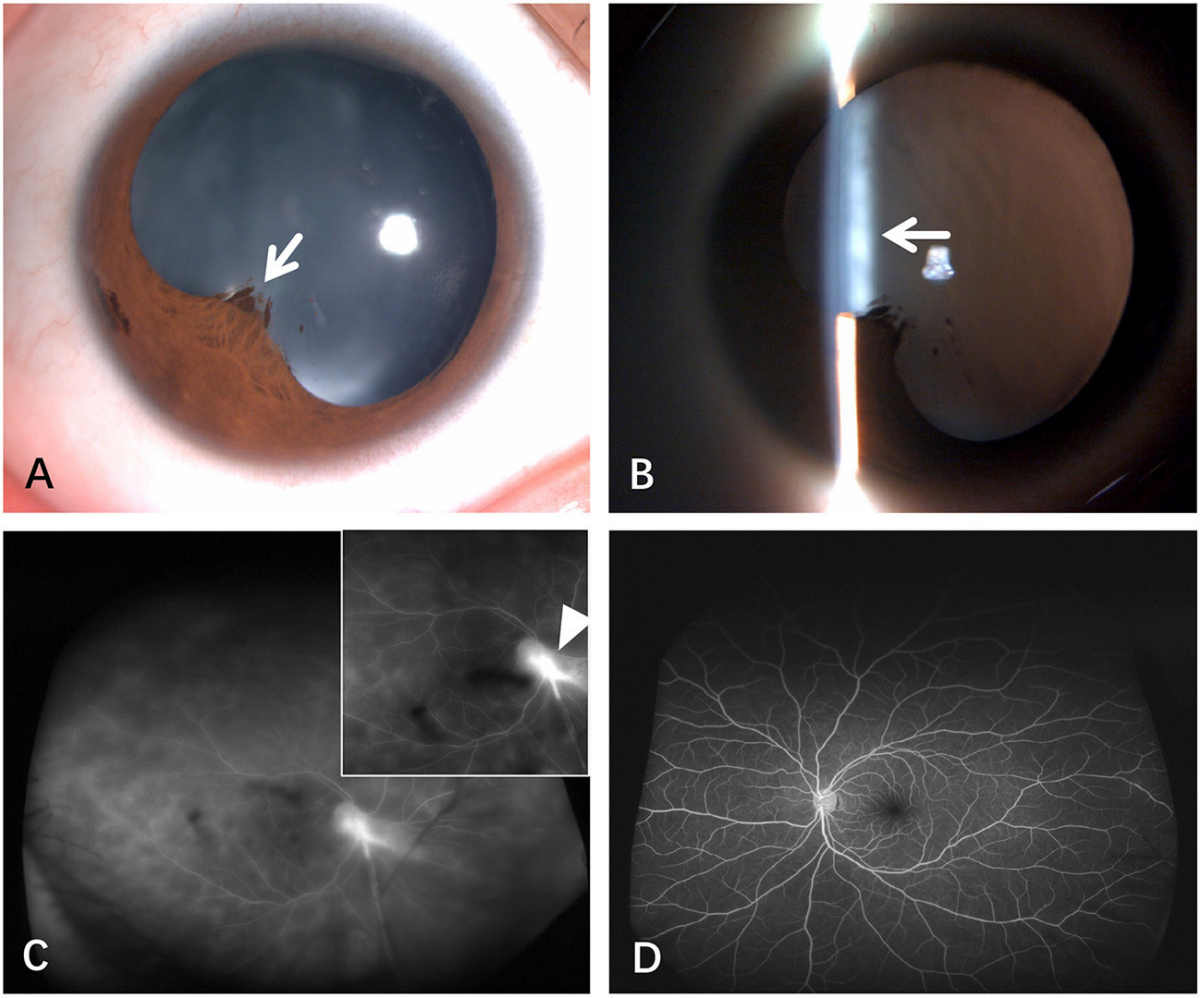
Anterior segment photography and FFA examination at the initial visit suggest OT. Using the diffuse illumination method, the posterior synechiae of the iris (white arrow) was visible with a slit lamp **(A)**. Cortical opacification of the lens (white arrow) was observed using a slit lamp with a narrow light band **(B)**. The refractive stroma was unclear, with a large area of subretinal hyperfluorescence and prepapillary membranous hyperfluorescence extending below the retina (white arrowhead). The small image in the upper right corner is the enlarged view of the posterior pole of the right eye **(C)**. FFA image of the left eye showed no retinal fluorescence leakage **(D)**.

**Figure 2:**
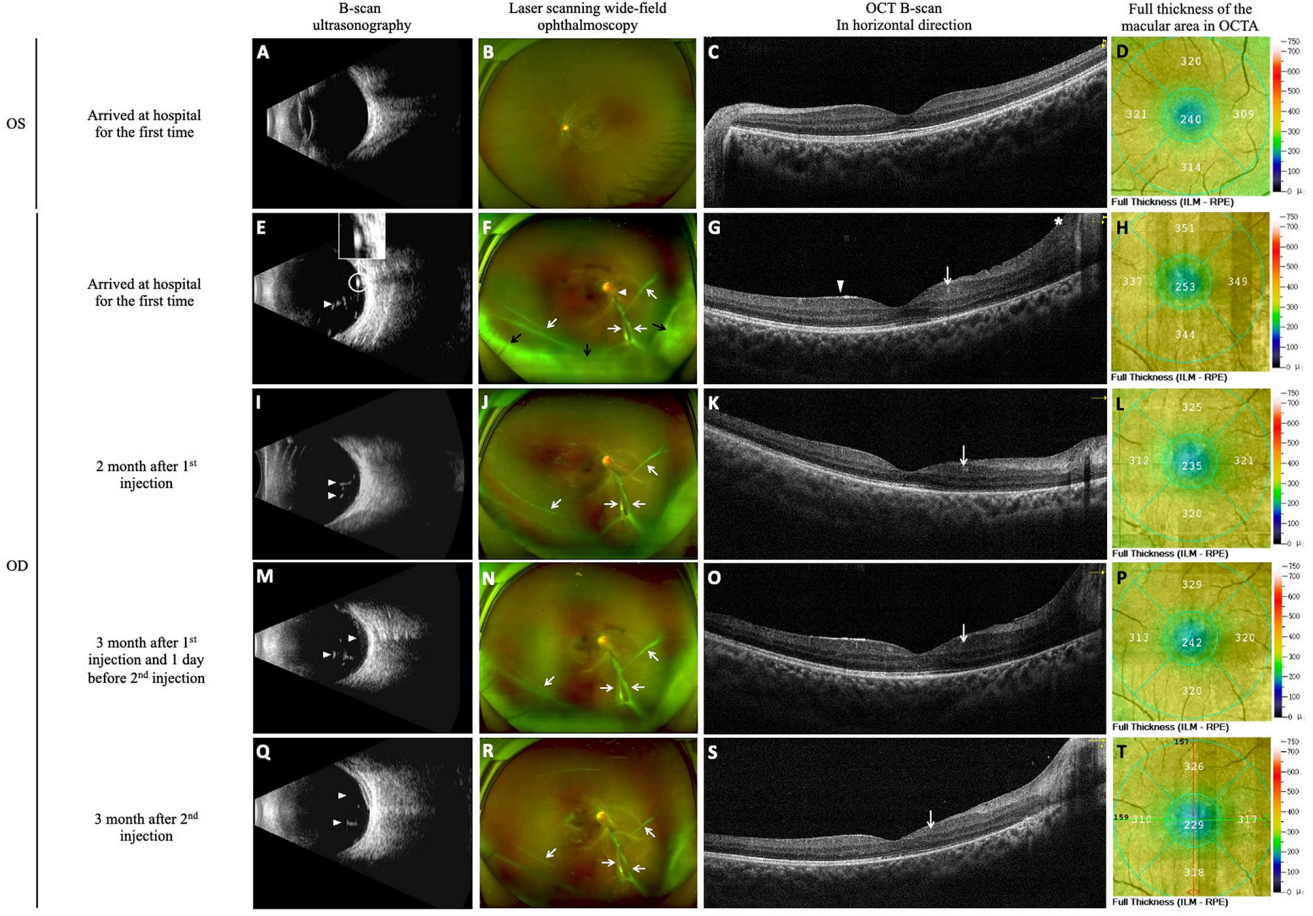
DEX implants were used to control uveitis. The images of B-scan ultrasound, laser-scanning wide-field ophthalmoscopy, macular OCT B-scan images and OCTA full-thickness macular retinal thickness images were obtained at initial diagnosis and follow-up. At the time of initial diagnosis, B-scan ultrasonography of the left eye showed clear vitreous cavity **(A)**, laser scanning wide-field ophthalmoscopy image showed clear optic disk boundaries, flat retina, and visible macular reflection in the left eye **(B)**, OCT B-scan image of the macular showed normal macular thickness **(C)**, and OCTA image showed a full-thickness macular foveal retinal thickness of 240 μm **(D)**. Examination of the right eye showed a reduction in vitreoretinal inflammation with DEX implant treatment. At initial diagnosis, a few light spots and strips were seen in the vitreous (white arrowhead), and a thick band of cord-like light adhering to the optic disc (white circle) was also observed **(E)**. Vitreous opacity was scored as 1+, the nasal and inferior borders of the optic disc were unclear in the right eye (white arrowhead), and a proliferative membrane anterior to the optic disc extends into the nasal and inferior retina (white arrows). The inferior peripheral retina is obscured by vitreous opacity (black arrows) **(F)**. Examination of the right eye showed increased full thickness of the macular area (full thickness of the macular fovea was 253 μm), coarse internal limiting and macular epiretinal membranes (white arrowhead), cystoid retina (white arrow), and thickened and elevated peripapillary retina (the asterisk) **(G, H)**. At re-examination 2 months after the first injection of DEX implant, the number of light spots in the vitreous (white arrowheads) decreased, indicating slightly reduced opacity **(I)**. Vitreous opacity was reduced and scored as Trace, and the proliferative membrane was unchanged (white arrows) **(J)**. The right eye showed decreased full thickness of the macular area (235 μm at the fovea), and the cystoid retina was still visible (white arrow) **(K, L)**. At re-examination 3 months after the first injection of DEX implant (the day before the second injection), more light spots were observed in the vitreous compared with the previous month (white arrowheads), indicating increased opacity **(M)**. The vitreous opacity score increased to 1+, and the proliferative membrane was unchanged (white arrows) **(N)**. The right eye showed a thicker full thickness of the retina compared with the previous month (242 μm at the fovea) and increased retinal cystoid change (white arrow) **(O, P)**. At re-examination 3 months after the second injection of DEX implant, the light spot in the vitreous (white arrowheads) decreased, indicating reduced opacity **(Q)**. Vitreous opacity was reduced and scored as Trace, and the proliferative membrane was unchanged (white arrows) **(R)**. The right eye showed thinning of the full thickness of the retina (229 μm at the fovea) and reduced retinal cystoid changes (white arrow) **(S, T)**.

Ophthalmic examination during the initial diagnosis showed uncorrected visual acuity of 20/400 in the right eye and 20/20 in the left eye, which could not be improved with corrected visual acuity. Intraocular pressure (IOP) was 15/12 mmHg (1 mmHg = 0.133 kPa). Both eyes had normal ocular alignment. Slit-lamp examination of the right eye revealed no posterior corneal deposits or aqueous flare; however, mydriatic examination revealed posterior synechiae (iris adhesions to the lens) occurring at the 6 to 8 o'clock positions, and lens opacity was observed (Figures 1A, B). Vitreous opacity was scored as 1+ (Nussenblatt scale), and gray-white mass opacifications were observed below the vitreous cavity. B-mode ultrasound examination showed hyperechoic spots and streaks in the vitreous, as well as hyperechoic cord-like thick light bands adhering to the optic nerve head (Figure 2E). Laser-scanning wide-field ophthalmoscopy showed unclear nasal and inferior boundaries of the optic disk, extension of the anterior proliferative membrane to the nasal 3 and 5 o'clock positions and inferotemporal region, and vitreous opacity obscuring the peripheral retina below the 3 to 9 o'clock positions (Figure 2F). Moreover, B-scan images of optical coherence tomography (OCT) revealed retinal thickening with cystoid changes, peripapillary retinal thickening and swelling, preretinal vitreous opacity granules, and epiretinal membrane, described as a rough internal limiting membrane (Figure 2G). Optical coherence tomography angiography (OCTA) revealed increased macular retinal thickness (full-thickness central macular retinal thickness, 253 μm) (Figure 2H). Fundus fluorescein angiography (FFA) showed extensive subretinal and epiretinal membranous hyperfluorescence in the late stage (Figure 1C). The patient's left eye showed normal examination findings (Figures 1D, 2A–D).

The patient was initially diagnosed with chronic uveitis, retinal vasculitis, and a complicated cataract in the right eye, with suspicion of peripheral granulomatous OT. Upon taking a medical history, his family reported that the patient had long-term close contact with dogs that were not administered with anthelmintics.

On July 11, 2021, he underwent aqueous humor aspiration of the right eye and aqueous humor metagenomic testing under general anesthesia. ELISA results showed increased anti-Toxocara IgG levels in serum and IF at 53.52 U (reference value < 9U) and 33.43 U (reference value < 3U), respectively. Additionally, the Goldmann–Witmer coefficient (GWC) of Toxocara was 24.82 (reference value < 2). The tests for other infectious, inflammatory, toxic, or paraneoplastic factors were also negative. Eventually, he was diagnosed with peripheral granulomatous OT in the right eye.

**Figure 3:**
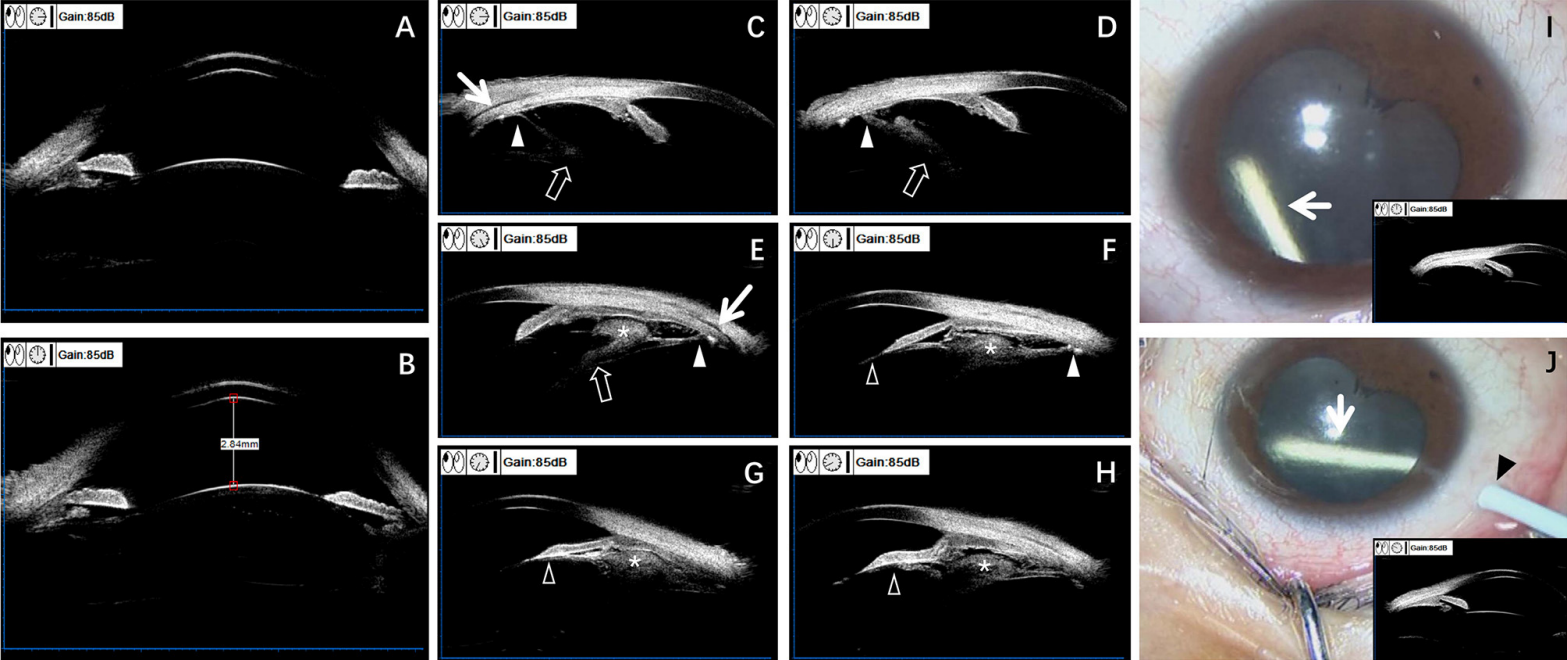
Preoperative UBM aids in diagnosing OT and guiding the selection of DEX implant injection sites. UBM examination images **(A–H)** and surgical video screenshots **(I, J)** of the right eye. Anterior segment at 3 o'clock and 9 o'clock positions **(A)**. Anterior segment at 12 o'clock and 6 o'clock positions **(B)**. Central anterior chamber depth ~2.84 mm. The iridocorneal angle is open in all directions. The distance from the ciliary process to the equator of the lens varies in different directions **(A, B)**. Examination after pupil dilation: moderate echo detected in the ciliary body and peripheral vitreous at ~3, 4, 5, 6, 7, and 8 o'clock positions **(C–H)**. Granulomas can be seen on the surface of the ciliary body (the asterisks) **(E–H)**, adhering to the ciliary body but with clear boundaries. The surrounding vitreous opacity strand attaches anteriorly to the lens and posterior iris (feint arrowhead) **(F–H)**, and posteriorly to the ciliary body at one attachment point (white arrowhead) **(C–F)**, causing mild tractional ciliary body detachment (white arrow) **(C, E)**. The vitreous opacity strand also extends toward the lens-vitreous direction (feint arrow) **(C–E)**. Based on preoperative UBM examination results, the anterior segment at the 10 o'clock and 12 o'clock positions appears normal **(I, J)**. Surgical video screenshots of the right eye intravitreal injection of DEX implants (white arrows) (two injections spaced 3 months apart) **(I, J)**. The first injection is performed at the 12 o'clock position **(I)**, and the second injection is performed at the 10 o'clock position (black arrow) **(J)**.

Prior to the definitive diagnosis, we performed UBM examinations on both eyes of the child. An experienced ultrasound physician used a UBM device (BME-300 MEDA, China) to examine the patient. Topical anesthesia was achieved with 1% tetracaine. The patient was positioned supine for the examination. An 18 mm diameter eye cup was placed in the conjunctival sac according to the size of the eyeball, and then filled with saline. Scans were performed on 12 endpoints of both eyes, with a focus on the pars plana, peripheral vitreous, and peripheral retina. The UBM examination showed that the central anterior chamber depth of the right eye was approximately 2.84 mm, with open angles in all directions. The distance from the ciliary process to the equator of the lens varied in different directions, with moderate to strong echoes in the ciliary body and surrounding vitreous at the 3 to 8 o'clock positions (Figures 3A–H). Upon further analysis of the UBM images, we found that peripheral granulomas appeared as irregular high-echo solid masses on the UBM, located on the surface of the ciliary body, with visible granulomas attached to the ciliary body but with a clear boundary. The surrounding vitreous strand extended anteriorly and attached to the posterior surface of the lens and iris, and posteriorly to the ciliary body, causing mild tractional ciliary body detachment. The vitreous strand was also observed extending in the direction of the lens and vitreous (Figures 3C–H).

Preoperative UBM examination indicated the presence of peripheral granulomas in the ciliary body and surrounding vitreous body from the 3 to 8 o'clock positions (Figures 3C–H). Moreover, laser-scanning wide-field ophthalmoscopy indicated the presence of epiretinal proliferative membrane in the posterior pole and peripheral retina from the 3 to 9 o'clock positions (Figure 2F). Therefore, the surgeon selected the 12 and 10 o'clock positions where there were no granulomas and peripheral vitreous strand for the subsequent two DEX implant injections, respectively, to avoid the granulomas and proliferative membranes, preventing injection-related complications (Figures 3I, J).

Before diagnosis, topical anti-inflammatory eye drops and mydriatic drugs were administered. After diagnosis, single-dose albendazole 400 mg was administered orally. On July 15, 2021, he underwent an intravitreal DEX implant injection in the right eye under general anesthesia (Figure 3I). The patient was followed up in our hospital monthly postoperatively to reexamine his eye condition. One and 2 months postoperatively, his visual acuity improved. Ophthalmic examination (2 months postoperatively) indicated that corrected visual acuity of the right eye improved to 20/100. B-ultrasound showed reduced vitreous opacity compared with that before the intervention (Figure 2I). Additionally, laser-scanning wide-field ophthalmoscopy also revealed reduced vitreous opacity scored as Trace (Nussenblatt scale) (Figure 2J). OCT B-scan showed retinal thickness thinning in the right eye after treatment, suggesting reduced edema (OCTA showed a full-thickness retinal thickness of the macular fovea at 235 μm) (Figures 2K, L). At 3 months postoperatively, the patient complained of slightly decreased visual acuity compared with the previous follow up. Ophthalmic examination showed decreased corrected visual acuity of the right eye at 20/200. B-ultrasonography and laser-scanning wide-field ophthalmoscopy revealed worsening vitreous opacity, which was scored as 1+ (Nussenblatt scale) (Figures 2M, N). OCT B-scan revealed increased edema (OCTA revealed the full-thickness retinal thickness of 242 μm in the central fovea) (Figures 2O, P). We speculated that the DEX implant was not sufficiently effective at 3 months postoperatively. Thus, a second intravitreal DEX implant injection was performed under general anesthesia on the next day (Figure 3J). At 1, 2, and 3 months after the second injection, his visual acuity gradually improved. At 3 months after the second injection, the corrected visual acuity improved to 20/50. B-ultrasonography and laser-scanning wide-field ophthalmoscopy showed reduced vitreous opacity, which was scored as Trace (Nussenblatt scale) (Figures 2Q, R). OCT B-scan revealed decreased retinal thickness and reduced retinal cystoid changes, suggesting reduced edema (OCTA showed the full-thickness foveal retinal thickness of 229 μm) (Figures 2S, T). Furthermore, laser-scanning wide-field ophthalmoscopy revealed stable proliferative membranes during the whole process (Figures 2E, J, N, R). One day after each of two injections, the IOP was slightly elevated, with values of 19/12 mmHg and 20/13 mmHg, respectively. We treated the patient with local IOP-lowering eye drops, carteolol hydrochloride ophthalmic solution (Mikelan), and at 1 month post-injection, the IOP returned to normal levels, with values of 14/12 and 15/13 mmHg, respectively.

Due to the COVID-19 pandemic, the patient cannot be followed up on schedule, and we recommended that he should be regularly examined at their local hospital. During a telephone follow-up 6 months after the second surgery, the patient reported stable vision in the right eye without any complications or adverse events.

**Figure 4:**
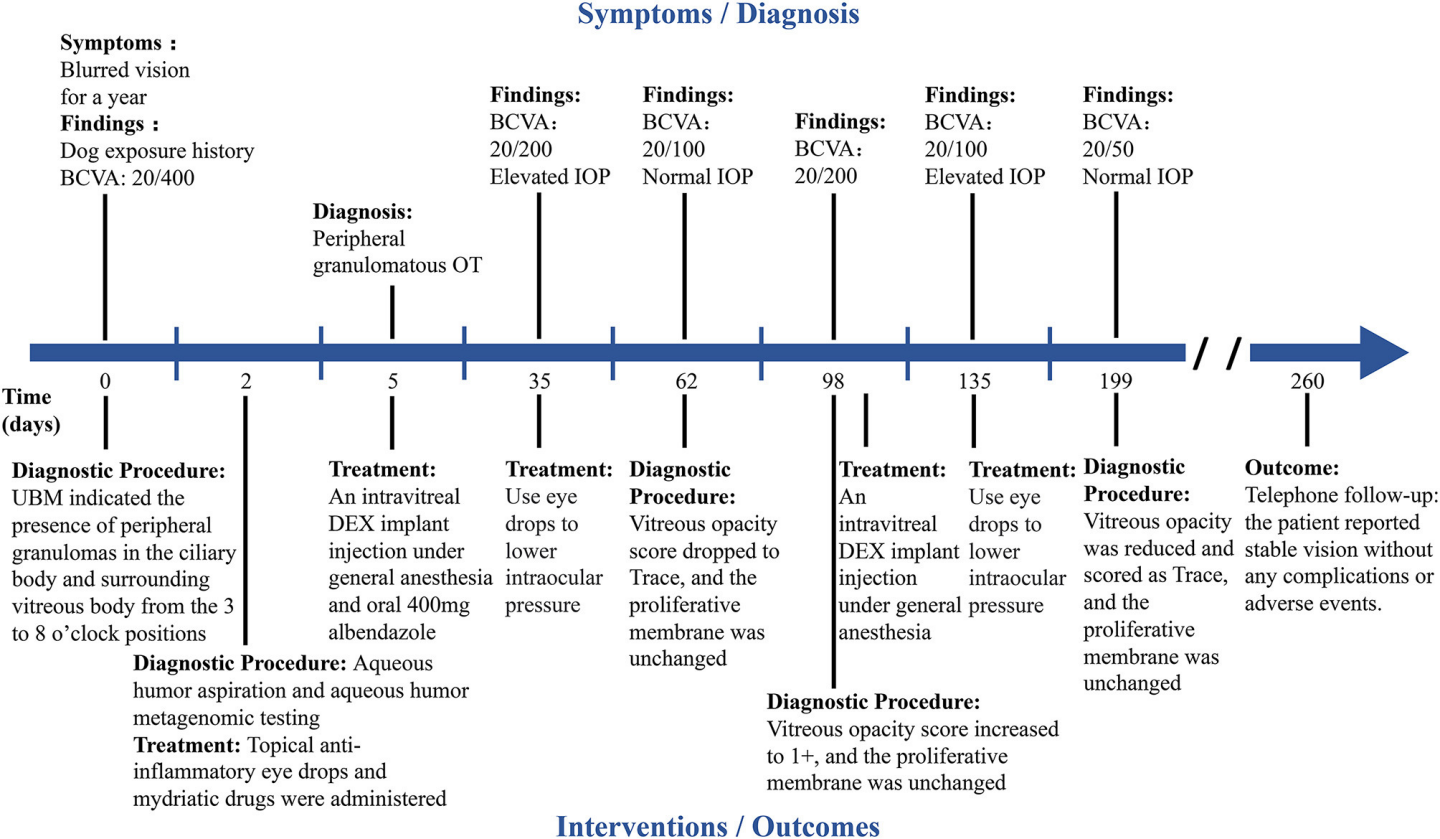
Timeline summarizing the progression of OT in the right eye. The key events or multimodal imaging examination results in the progression of OT are indicated by short lines along the timeline.

During the treatment period, the patient did not take any oral steroids. No other adverse events related to the local application of DEX implants, such as corneal edema, rapid cataract progression, retinal breaks, retinal detachment, or intraocular infection, were observed (Figure 4).

## 3. Discussion

OT is a highly neglected zoonotic disease that mainly affects the unilateral eye of children and adolescents (4). The overall seropositivity rate for Toxocara antibody is 19.3% in Chinese children, which should draw the government's attention (29). The cognition of children is worse than that of adults, and their eye diseases often cannot be diagnosed promptly, leading to irreversible vision loss even with aggressive treatment. In our case, the patient was an 8-year-old child who complained of blurred vision for 1 year, with poor visual acuity at the initial diagnosis. The child had a history of long-term contact with dogs, and ophthalmic examinations indicated chronic uveitis. UBM suggested the presence of peripheral granuloma, and elevated levels of IgG and GWC in serum and IF anti-Toxocara ELISA supported the diagnosis of peripheral granulomatous OT. The child in our case did not meet the indications for vitrectomy. The primary goal of treatment was to alleviate uveitis, improve visual prognosis, and prevent complications. We employed UBM-guided intravitreal injection of DEX implants, which controlled the uveitis and improved the vision from 20/400 to 20/50.

UBM examination has significant value in the diagnosis of OT and provides guidance for the selection of injection sites for DEX implants. Since ~80% of granulomas in peripheral granulomatous OT are found on the ciliary body surface, which is difficult to observe in routine clinical examinations, UBM provides valuable supplementary information. Compared to conventional ultrasound or color Doppler imaging (CDI), UBM has higher spatial resolution and is suitable for detailed examination of the anterior segment. The value of indirect ophthalmoscopy is limited due to the blurring of anterior segment structures caused by the turbidity of the refractive media in the anterior segment and peripheral retina (25). UBM has certain value in the diagnosis and differential diagnosis of peripheral granulomatous OT. Its application has confirmed the existence of mixed-type (with both peripheral and posterior pole granulomas) OT, and has improved the detection rate of peripheral granulomas (27). UBM images show that peripheral granulomas are olive-shaped in the radial position and rod-shaped in the coronal position. This characteristic morphology helps differentiate OT from other peripheral diseases, such as intermediate uveitis and granulomas induced by ciliary body inflammation (27). In addition, preoperative UBM examination can determine the location and extent of peripheral granuloma, which is an advantage over preoperative fundus examination and intraoperative indentation examination (18, 27). Therefore, UBM examination can guide surgeons in selecting more appropriate surgical or injection entry points to reduce the risk of peripheral granuloma rupture or tractional retinal detachment and prevent serious surgical complications (18, 27). In the case of our patient, UBM examination revealed granulomas on the ciliary body surface from 3 to 8 o'clock positions, and the surrounding vitreous strand was partially adherent to the lens and ciliary body, causing mild ciliary body detachment, indicating that inflammation should be stabilized as soon as possible to avoid peripheral tractional retinal detachment. Moreover, laser scanning wide-field ophthalmoscopy showed an epiretinal proliferative membrane in the posterior pole and surrounding retina from the 3 to 9 o'clock positions. Based on the above findings, the surgeon ultimately chose to perform two intravitreal injections at the 12 and 10 o'clock positions in the pars plana. No retinal damage was observed postoperatively. Furthermore, we found that the clinical significance of the vitreous opacity strands surrounding the granuloma is not yet clear, and similar lesions can occur in patients with necrotizing scleritis, intermediate uveitis, and post-cryotherapy scleral melting. It can be hypothesized that they may be relatively consistent responses of the vitreous to inflammation. In summary, UBM examination has important significance in the diagnosis, differential diagnosis, and preoperative evaluation of OT. It not only helps to discover and differentiate peripheral granulomas, but also provides a basis for surgeons to select more appropriate surgical or injection entry points. Additionally, the ability of UBM to evaluate postoperative anterior segment conditions following DEX implant treatment for OT warrants further clinical investigation.

DEX intravitreal sustained-release implant is a biodegradable ophthalmic drug that slowly releases 0.7 mg of DEX to inhibit inflammation, reducing patients' reliance on systemic steroids and immunosuppressants (30–32). Existing research has demonstrated that DEX implant injections can improve vitreous opacity in patients with OT and also improve visual prognosis in cases where the macula is not involved (33). Both children and post-PPV eyes have shown effectiveness with repeated DEX implant injections (14, 17, 34, 35). In our case, the patient's vision improved, and inflammation was controlled after the injection. Oral steroids were not administered during treatment.

Several issues may be encountered during the treatment of OT with DEX implants. (1) Increased intraocular pressure (IOP) post-injection: DEX injection can cause ocular hypertension (OHT) (IOP ≥ 25 mmHg and/or an increase of ≥10 mmHg compared to baseline), which is attributable to the DEX implant itself. However, IOP can be controlled in the short term with the use of some topical IOP-lowering medications (33, 36–38). In our case, the patient experienced elevated IOP after injection, but it returned to pre-injection levels after the use of IOP-lowering eye drops. A decrease in IOP may also indicate a weakening of the drug's effect. (2) Rapid progression of cataract post-injection: The use of local DEX may cause rapid progression of cataract. However, studies have found no significant differences between the DEX implant treatment group and the control group in the progression and formation of cataracts (33). Furthermore, OT itself may also contribute to cataract progression. Our patient had mild cortical opacification of the lens before treatment, and there was no progression of cataract after treatment, with improved vision. (3) Timing of retreatment: DEX implant has biphasic pharmacokinetics, releasing a high dose of DEX initially, peaking at day 60, followed by a rapid decrease in concentration between days 60 and 90, then stabilizing at a lower concentration until day 180. Its therapeutic effect generally lasts no more than 6 months. Studies on the use of DEX implants for the treatment of retinal vein occlusion (RVO) or non-infectious uveitis have shown that a retreatment interval of 3 to 5 months is sufficient to maintain efficacy (39). Research on the use of DEX implants for the treatment of OT found that the average interval for retreatment in patients with recurrent inflammation was 3.7 ± 1.0 months (median of 3 months) (33). The timing of the re-injection of DEX implant for treating OT is still controversial. Patients need to be followed closely every month and receive another DEX injection before the onset of active inflammation. The method for determining the timing of retreatment is still unresolved. We believe that relying solely on a decrease in IOP, deterioration of vision, or increased vitreous opacity cannot assess the timing of retreatment, as active inflammation usually occurs before these changes. In our case, the child experienced decreased vision, increased vitreous opacity, and re-activation of inflammation at the 3-month follow-up, indicating that the drug's effect had weakened after 3 months and necessitating another DEX implant injection. At 1, 2, and 3 months post-second injection, the patient's vision improved significantly, and vitreous inflammation was reduced. Despite the occurrence of elevated IOP, the treatment outcome in this case was still satisfactory.

Traditional treatment methods for OT include pharmacological therapy and surgical intervention. Although long-term oral steroids combined with albendazole can reduce the recurrence rate within 6 months, prolonged use of oral steroids may lead to drug-induced glaucoma and cataract. The loss of vision remains irreversible even after further cataract or anti-glaucoma surgery (40). For children, long-term systemic use of steroid drugs can affect the growth and development of the body. Inflammation may recur upon steroid reduction or withdrawal, requiring patients to resume steroids use or increase the dosage (29, 41). The indications for PPV are epiretinal membrane, tractional or rhegmatogenous retinal detachment, and severe or persistent vitreous opacity. However, PPV alters the intraocular tamponade and affects the eye's internal structures, potentially leading to complications such as elevated IOP, rapid cataract progression, retinal detachment, and intraocular infection, resulting in a poor postoperative visual prognosis. For children, additional factors must be considered. The child in this case was diagnosed with peripheral granulomatous OT, characterized by mild to moderate vitreous opacity and an unaffected macula. The primary goal of treatment was to alleviate uveitis and prevent complications such as tractional retinal detachment induced by OT. Therefore, we did not consider performing PPV and chose intravitreal injection of DEX implants as an alternative to long-term systemic steroid therapy for anti-inflammatory treatment.

## 4. Conclusion

UBM can accurately determine the location and extent of peripheral granulomas in OT patients, facilitating the avoidance of granulomas during intravitreal injection and preventing complications associated with intravitreal injection. For the treatment of pediatric patients with peripheral granulomatous OT, we recommend adopting a treatment plan of intravitreal injection of DEX implants guided by UBM. With close follow-up and strict adherence to indications, this approach can effectively alleviate uveitis and improve visual prognosis while eliminating the need for long-term systemic administration of any steroids. This case report emphasizes that the treatment strategy combining UBM and DEX implants can provide an effective and safe treatment option for children with peripheral granulomatous OT.

## Data availability statement

The original contributions presented in the study are included in the article/supplementary material, further inquiries can be directed to the corresponding author.

## Ethics statement

The studies involving human participants were reviewed and approved by Ethic Committee of the Second Affiliated Hospital of Zhejiang University. Written informed consent to participate in this study was provided by the participants' legal guardian/next of kin. Written informed consent was obtained from the individual(s), and minor(s)' legal guardian/next of kin, for the publication of any potentially identifiable images or data included in this article.

## Author contributions

ZZ provided this case and offered guidance. XZ performed the data analysis and wrote the manuscript. XH, YZ, and JL contributed to discussion. All authors contributed to the article and approved the submitted version.
